# Accuracy of the Huawei GT2 Smartwatch for Measuring Physical Activity and Sleep Among Adults During Daily Life: Instrument Validation Study

**DOI:** 10.2196/59521

**Published:** 2024-12-20

**Authors:** Longfei Mei, Ziwei He, Liang Hu

**Affiliations:** 1 Department of Sports Science, College of Education, Zhejiang University, No. 866, Yuhangtang Road, Hangzhou, 310030, China, 86 18667127699; 2 Digital Sports and Health Laboratory, College of Education, Zhejiang University, Hangzhou, China

**Keywords:** smartwatch, accelerometry, free-living, physical activity, sleep, validity

## Abstract

**Background:**

Smartwatches are increasingly popular for physical activity and health promotion. However, ongoing validation studies on commercial smartwatches are still needed to ensure their accuracy in assessing daily activity levels, which is important for both promoting activity-related health behaviors and serving research purposes.

**Objective:**

This study aimed to evaluate the accuracy of a popular smartwatch, the Huawei Watch GT2, in measuring step count (SC), total daily activity energy expenditure (TDAEE), and total sleep time (TST) during daily activities among Chinese adults, and test whether there are population differences.

**Methods:**

A total of 102 individuals were recruited and divided into 2 age groups: young adults (YAs) and middle-aged and older (MAAO) adults. Participants’ daily activity data were collected for 1 week by wearing the Huawei Watch GT2 on their nondominant wrist and the Actigraph GT3X+ (ActiGraph) on their right hip as the reference measure. The accuracy of the GT2 was examined using the intraclass correlation coefficient (ICC), Pearson product-moment correlation coefficient (PPMCC), Bland-Altman analysis, mean percentage error, and mean absolute percentage error (MAPE).

**Results:**

The GT2 demonstrated reasonable agreement with the Actigraph, as evidenced by a consistency test ICC of 0.88 (*P*<.001) and an MAPE of 25.77% for step measurement, an ICC of 0.75 (*P*<.001) and an MAPE of 33.79% for activity energy expenditure estimation, and an ICC of 0.25 (*P*<.001) and an MAPE of 23.29% for sleep time assessment. Bland-Altman analysis revealed that the GT2 overestimated SC and underestimated TDAEE and TST. The GT2 was better at measuring SC and TDAEE among YAs than among MAAO adults, and there was no significant difference between these 2 groups in measuring TST (*P*=.12).

**Conclusions:**

The Huawei Watch GT2 demonstrates good accuracy in step counting. However, its accuracy in assessing activity energy expenditure and sleep time measurement needs further examination. The GT2 demonstrated higher accuracy in measuring SC and TDAEE in the YA group than in the MAAO group. However, the measurement errors for TST did not differ significantly between the 2 age groups. Therefore, the watch may be suitable for monitoring several key parameters (eg, SC) of daily activity, yet caution is advised for its use in research studies that require high accuracy.

## Introduction

Physical inactivity is recognized as a significant risk factor for many chronic noncommunicable diseases, including cardiovascular disease, certain malignancies (eg, colorectal cancer), and type 2 diabetes [1], leading to increased mortality, reduced quality of life, shorter life expectancy [2-4], and a significant financial burden on the health care system [5]. Hence, interventions aimed at increasing physical activity are critical to public health. In this regard, sustained efforts of regular and accurate monitoring of physical activity are necessary [6-8]. To achieve this goal, researchers and health practitioners have been continuously in search of physical activity measurement methods that best balance precision and cost. High-precision monitoring methods such as double-labeled water and indirect calorimetry are not suitable for daily life due to high cost and user burden [9]. Pedometers and accelerometers are relatively easy to carry in daily life but offer only limited indices that mostly focus on activity counts, rather than physiological measures (eg, heart rate), and the latter is also expensive [8,9]. Questionnaires are commonly used, particularly in large-sample surveys, but have often been questioned for being highly subjective [8-10].

With rapid technological advancements, smartwatches have become increasingly popular for monitoring and promoting health behaviors such as physical activity, sedentary behavior, and sleep. Such devices are valued for their multifunctionality, convenience, comfort, and cost-effectiveness. Specifically, smartwatches have multiple built-in sensor devices, such as accelerometers and optical heart rate sensors, which may achieve a higher level of measurement accuracy and cover a variety of indices (eg, duration of certain behavior, heart rate, and oxygen saturation) than pedometers and accelerometers that are commonly used in research settings [11].

Recent studies highlight the role of smartwatches in enhancing exercise motivation and facilitating healthier lifestyle choices [12-15]. With the growing popularity, smartwatches provide the possibility for continuous, real-time monitoring and feedback, which have been shown effective in increasing physical activity engagement [7,12-16]. Nowadays, various types of smartwatches have been widely used in a variety of practical settings, such as physical activity programs, fitness training, physical education, and health care services [7,17-19]. They also have great potentials to serve research purposes [20,21]. However, the practical applications of smartwatches (eg, in fitness training, physical education, and health care services) as well as their use in research should rely on continuous validation and improvement of their accuracy.

Several studies have evaluated the accuracy of various smartwatch models in real-world settings, comparing them against established activity monitoring devices such as the Accelerometer GT3X+ [22,23]. Degroote et al [22] observed that while models such as the Polar M600 and Huawei Watch accurately measured steps, their ability to accurately capture moderate to vigorous physical activity varies. Tedesco et al [23] found that for older adult population, both the Fitbit Charge 2 and the Garmin Vivosmart HR+ were effective in step counting but only moderately accurate in measuring activity energy expenditure and sleep, with Fitbit performing slightly better in these areas. In addition, several studies examined the accuracy of smartwatches in different age groups in laboratory setting [24,25]. Chow and Yang [24] found that when performing aerobic exercise indoors, the Xiaomi Mi Band 2 and Garmin Vivosmart HR+ showed overall acceptable accuracy in heart rate measurements, which were independent of age. Magistro et al [25] suggested that the ADAMO Care Watch, with its algorithm tailored for slower pace, might be particularly effective for older adults in step counting.

While the accuracy of smartwatches has been explored in previous research studies, research specifically focusing on their performance across different age groups in a free-living environment remains scarce. The Huawei smartwatch ranks second in global sales in Q2 2023 and holds a high market share of 39% in China [26] but received limited empirical research attention on its accuracy [22,27], especially with regard to measurements of activity energy expenditure and sleep duration [28-30]. Considering its significant market prominence, it is crucial to rigorously assess the accuracy of the Huawei smartwatch in measuring health-related indices to ensure its validity for users’ health monitoring and lifestyle management. Clearly, researchers have long voiced out the need to regularly examine the accuracy of smartwatches in order to continuously improve their functions and better apply them to human health promotion. However, it appears that studies of validity examination are far from providing sufficient evidence that support the use of smartwatches for research and health promotion purposes [23,28,29,31].

Hence, this study aimed to determine the validity of Huawei Watch GT2 for different populations under free-living conditions. Such efforts aimed to address the lack of evidence regarding the device’s performance across diverse user groups and provided insights that may have facilitated the broader adoption of smartwatches in health promotion and monitoring.

## Methods

### Participants

Sample size estimation was performed using G*Power 3.1 (effect size *f*²=0.30, α=.05, and power=0.80), resulting in a required sample size of 84. A total of 120 adults were recruited through campus and community flyers. Eighteen participants opted out, claiming interference with their daily life and sleep caused by the device. Ultimately, data were retained for 102 participants, including 44 young adults (YAs; age range: 18‐24 years) and 58 middle-aged and older (MAAO) adults (age range: 55‐91 years). All the participants were Chinese, living in Hangzhou, China, and volunteered to participate in this study. The inclusion criteria were no neurological or cardiovascular disease that could affect the participants’ daily exercise and no history of lower extremity injury or disability. A detailed verbal explanation was provided by the researchers before participation in the study; all participants signed informed consent. Participants had the right to choose to continue or discontinue their participation at any time during the conduct of this experiment.

### Measures

#### Accelerometer

The Actigraph GT3X+ (ActiGraph) has been widely used as a reference measure of physical activity because it has been consistently found to be both reliable and valid in physical activity studies [32-34]. The Actigraph accelerometer was set to collect data every 10 seconds, and the raw data and clinical reports of the accelerometer were downloaded using ActiLife software (version 6.13.4; ActiGraph). The Freedson Adult (1998) cut points were used to process the data and calculate activity energy expenditure [35-37]. The total sleep time (TST) was calculated using the Cole-Kripke algorithm [38].

#### Wearable Devices

The study aimed to examine the validity of the then-latest Huawei Watch GT2 (45.9×45.9×10.7 mm), developed by Huawei Technologies Co, in daily life. The GT2 had a wrist coverage of 14‐21 cm and was equipped with the wearable chip Kirin A1 and sensors, including an acceleration sensor, a gyroscope, an optical heart rate sensor, and a geomagnetic sensor. At the time of the study, the device was capable of measuring activity energy expenditure, step count (SC), heart rate, and sleep monitoring. In this study, the researcher used Huawei Sports Health software (Huawei Technologies Co) for data acquisition, which was conducted in 2021, to examine the performance of the GT2 in terms of SC, total daily activity energy expenditure (TDAEE), and TST in daily life.

### Data Collection Procedure

The researchers measured and collected participants’ physiological data (age, gender, height, and weight) on the first day using mechanical height gauges and electronic scales and then fitted them with a calibrated Actigraph GT3X+ (right hip) and Huawei Watch GT2 (nondominant wrist). Participants were told to maintain a normal daily routine but to wear both devices for 7 consecutive days (including 2 weekends and 5 workdays). The devices were worn at all times, except during water-based activities.

### Statistical Analysis

The data were downloaded and recorded once the devices were returned. To ensure that the collected data were reliable, the researchers examined the data and excluded cases where the accelerometer was worn for less than 10 hours in a day and for fewer than 2 days [39,40]. Data analysis and graphical representation were performed using IBM SPSS 29 (IBM Corp) and GraphPad Prism 9 (GraphPad). First, a descriptive analysis of the GT2 and GT3X+ accelerometers was performed, and the Shapiro-Wilk test showed that the collected data followed a normal distribution (*P*>.05). Second, the correlations between GT2 and accelerometer data were examined by calculating the Pearson product-moment correlation coefficient (PPMCC) and the intraclass correlation coefficient (ICC; 2-way random, absolute consistency; 95% CI; and single measurement). The critical values for the explanatory ICC were categorized as poor for <0.60, moderate for 0.60-0.75, good for 0.75-0.90, and excellent for >0.90 [41]. Third, the agreement between GT2 and accelerometer was examined by calculating the mean percentage error (MPE) and the mean absolute percentage error (MAPE). The MPE quantifies the average percentage deviation between the GT2 and accelerometer, highlighting potential systematic discrepancies. Moreover, MAPE captures the absolute extent of these deviations, offering a comprehensive view of error magnitude without directional bias (the formula is: MAPE = (1/n) × Σ(|(Actual value − Predicted value)|/Actual value) × 100%). These metrics are instrumental in assessing the GT2’s precision across various physical activities. To determine whether the measurement errors of the GT2 against the Actigraph were statistically different between the 2 age groups, two-tailed independent *t* tests were used. Finally, to test the level of consistency between GT2 and convergence measures, Bland-Altman plots and the associated consistency restrictions were constructed. To intuitively display the accuracy of different indicators of the Huawei Watch GT2, Bull’s-eye diagrams based on the Actigraph accelerometer were constructed.

### Ethical Considerations

This study involving human participants was rigorously reviewed and approved by the ethics committee of the Department of Psychology and Behavioral Sciences, Zhejiang University ([2021]007). All study procedures adhered to the 1964 Declaration of Helsinki and its subsequent amendments, ensuring compliance with ethical standards. Written informed consent was obtained from all participants, with strong measures in place to safeguard personal data and privacy.

## Results

### Overview

A total of 102 participants met the inclusion criteria and participated in the experiment, spanning an age range of 18-91 (mean 48.17, SD 24.56) years and a BMI range of 16.68-31.64 (mean 22.52, SD 3.13) kg/m². The cohort was stratified into 2 age groups: YAs and MAAO adults, with a note that 5 participants in the MAAO group were younger than 60 years, constituting 8.62% (5/58) of this group. Detailed demographic data are shown in Table 1.

**Table 1.** Participant characteristics (N=102).

Characteristic	Total (N=102)	YAs^a^ (n=44)	MAAO^b^ (n=58)
Age (year), mean (SD)	48.17 (24.56)	20.89 (1.66)	68.86 (7.55)
Male gender, n (%)	37 (36.27)	22 (50)	15 (25.86)
Height (cm), mean (SD)	164.51 (8.57)	170.59 (7.38)	159.91 (6.25)
Weight (kg), mean (SD)	60.88 (9.07)	60.82 (8.53)	60.93 (9.54)
BMI (kg/m^2^), mean (SD)	22.52 (3.13)	20.86 (2.14)	23.78 (3.19)

a YA: young adult.

b MAAO: middle-aged and older.

**Figure 1.**
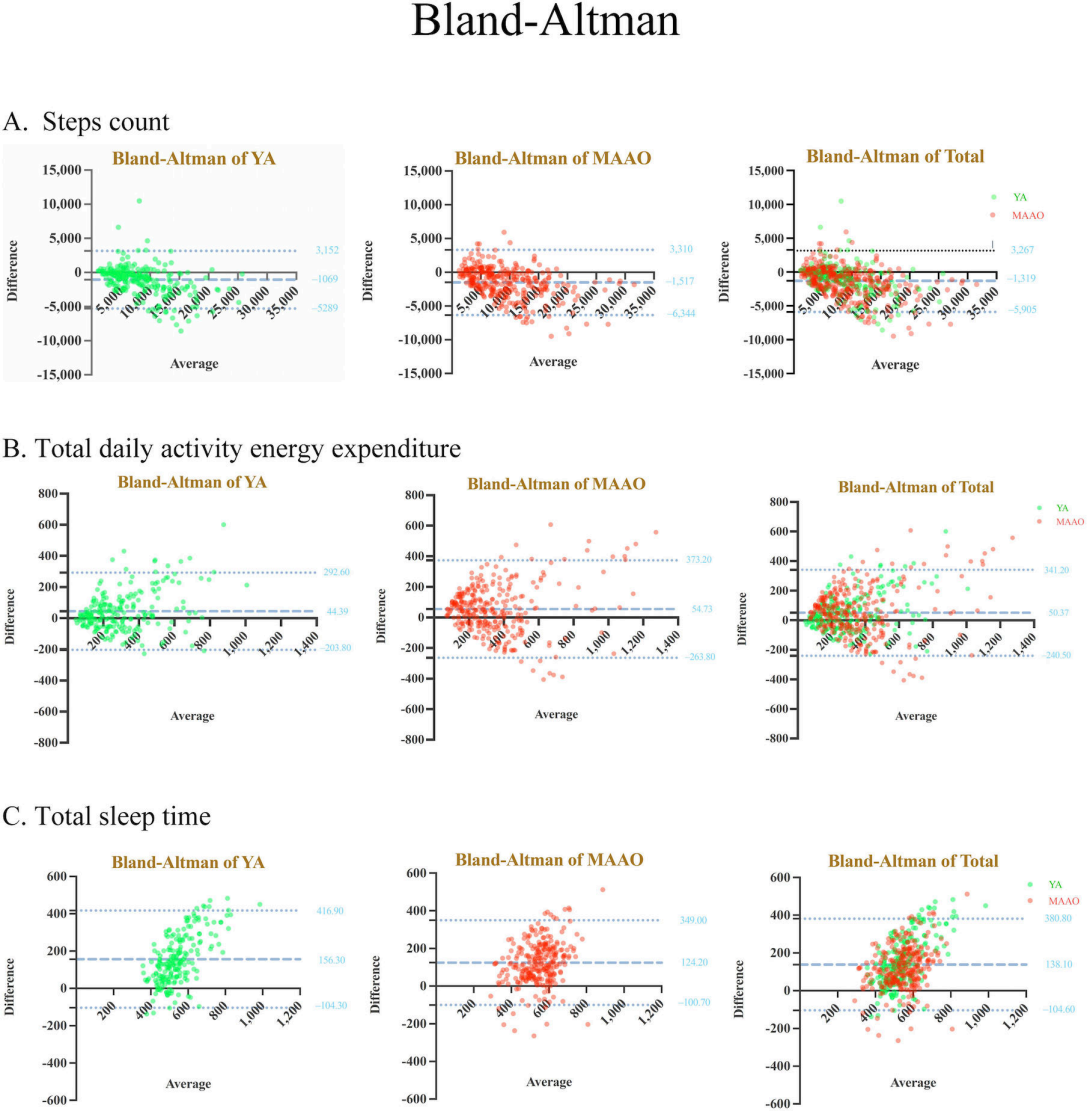
Bland-Altman plots of the Huawei Watch GT2 and Actigraph GT3X+ in overall phases for different groups and indicators. The middle line represents the mean difference between the GT2 and accelerometer (negative values indicate an overestimation of the GT2, whereas positive values indicate an underestimation), and the upper and lower dashed lines indicate the limit of agreement (1.96 × SD of the difference scores). The green dots indicate YAs, and the red dots indicate MAAOs. Panel A displays the Bland-Altman plot for step count (steps) across different groups, panel B presents the Bland-Altman plot for total daily activity energy expenditure (kcal) across different groups, and panel C illustrates the Bland-Altman plot for total sleep time (minutes) across different groups. MAAO: middle-aged and older adults; YA: young adults.

**Figure 2.**
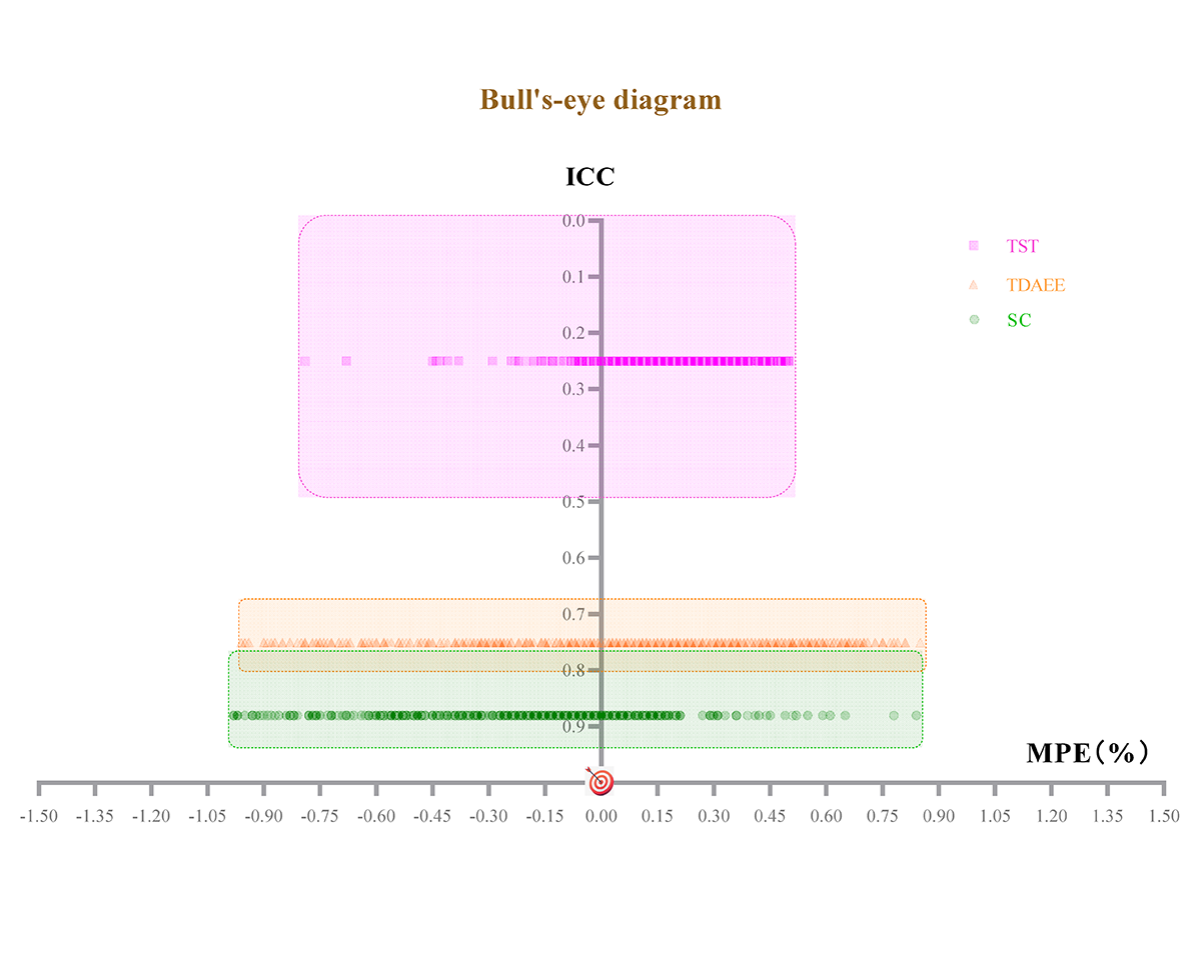
Bull’s-eye diagrams with Actigraph GT3X+ as the reference measure. The x-axis represents the MPE, and the y-axis represents the ICC between the GT2 and the accelerometer. The good accuracy of the Huawei Watch GT2’s measurement is indicated when the scatter point falls steadily near the origin (MPE=0, ICC=1). ICC: intraclass correlation coefficient; MPE: mean percentage error; SC: step count (steps); TDAEE: total daily activity energy expenditure (kcal); TST: total sleep time (minutes).

Table 2 shows the results of correlational analysis between the GT2 and the GT3X+ accelerometer, while Table 3 shows the results of the difference test between the GT2 and Actigraph in terms of SC, TDAEE, and TST during daily life across different age groups, along with the Bland-Altman limits of agreement. Figure 1 visualizes the Bland-Altman analysis comparison plot, and Figure 2 illustrates the overall accuracy of the Huawei Watch GT2 across various functions.

**Table 2.** Correlation coefficients, 95% CIs between Huawei Watch GT2 and Actigraph GT3X+ measurements, and independent sample *t* tests on mean absolute percentage error between the YA^a^ and MAAO^b^ groups.

Indicator, group	Correlation		Correlation		Independent	
	PPMCC^c^ (95% CI）	*P* value	ICC^d^ (95% CI）	*P* value	*t* test (*df*)^e^	*P* value
SC^f^					−3.45 (571)	<.001
Total	0.92 (0.91 to 0.93)	<.001	0.88 (0.76 to 0.93)	<.001		
YAs	0.91 (0.89 to 0.93)	<.001	0.88 (0.79 to 0.92)	<.001		
MAAO	0.92 (0.90 to 0.94)	<.001	0.88 (0.73 to 0.93)	<.001		
TDAEE^g^					−6.70 (512)	<.001
Total	0.77 (0.74 to 0.80)	<.001	0.75 (0.67 to 0.80)	<.001		
YAs	0.80 (0.75 to 0.84)	<.001	0.76 (0.67 to 0.83)	<.001		
MAAO	0.76 (0.71 to 0.80)	<.001	0.73 (0.65 to 0.80)	<.001		
TST^h^					1.54 (483)	.12
Total	0.47 (0.40 to 0.54)	<.001	0.25 (−0.06 to 0.49)	<.001		
YAs	0.49 (0.38 to 0.59)	<.001	0.21 (−0.07 to 0.46)	<.001		
MAAO	0.48 (0.39 to 0.57)	<.001	0.29 (−0.05 to 0.54)	<.001		

a YA: young adult.

b MAAO: middle-aged and older.

c PPMCC: Pearson product-moment correlation coefficient.

d ICC: intraclass coefficient correlation. <0.60 (poor), 0.60-0.75 (moderate), 0.75-0.90 (good), and >0.90 (excellent).

e Independent *t* test: independent samples *t* test between YA and MAAO group mean absolute percentage errors.

f SC: step count.

g TDAEE: total daily activity energy expenditure.

h TST: total sleep time.

**Table 3.** Measured mean, mean percentage error (MPE), mean absolute percentage error (MAPE), and deviation values in Bland-Altman analysis for the Huawei Watch GT2 and Actigraph GT3X+ accelerometer for step count (SC; steps), total daily activity energy expenditure (TDAEE; kcal), and total sleep time (TST; minutes), with associated SDs.

Indicator, group, device	Mean (SD）	MPE (SD)	MAPE (SD）	Bland-Altman analysis^a^, bias (SD)
SC				
Total		−17.47 (30.56)	25.77 (23.98)	−1318.82 (2340)
Watch GT2	9585.29 (5947.50)			
Accelerometer	8266.47 (4919.71)			
YAs^b^		−14.55 (27.80)	21.91 (22.43)	−1068.63 (2153)
Watch GT2	8870.82 (5333.84)			
Accelerometer	7802.18 (4418.34)			
MAAO^c^		−19.79 (32.44)	28.81 (24.74)	−1516.62 (2463)
Watch GT2	10,150.17 (6342.52)			
Accelerometer	8633.55 (5260.39)			
TDAEE				
Total		8.43 (39.64)	33.79 (22.34)	50.36 (148.40)
Watch GT2	307.76 (204.37)			
Accelerometer	358.13 (232.90)			
YAs		4.48 (36.94)	30.32 (21.48)	44.39 (126.60)
Watch GT2	282.41 (172.53)			
Accelerometer	326.80 (212.99)			
MAAO		11.32 (41.33)	36.29 (22.68)	54.73 (162.50)
Watch GT2	326.27 (223.26)			
Accelerometer	381.00 (244.24)			
TST				
Total		19.98 (18.20)	23.29 (13.71)	138.08 (123.80)
Watch GT2	476.68 (88.45)			
Accelerometer	614.76 (138.20)			
YAs		22.13 (17.23)	24.39 (13.84)	156.33 (133.00)
Watch GT2	466.60 (77.06)			
Accelerometer	622.94 (153.17)			
MAAO		18.35 (18.77)	22.45 (13.58)	123.15 (114.70)
Watch GT2	484.37 (95.68)			
Accelerometer	608.52 (125.51)			

a Bland-Altman analysis: bias of Bland-Altman.

b YA: young adult.

c MAAO: middle-aged and older.

### Step Count

First, Table 2 shows the correlation between the GT2 and the accelerometer, with a PPMCC test score of 0.92 (*P*<.001; 95% CI 0.91-0.93) and an ICC test score of 0.88 (*P*<.001; 95% CI 0.76-0.93), which suggests that the GT2 possesses good to excellent validity in measuring SC. Second, the difference between the GT2 and Actigraph in SC across age groups is shown in Table 3. Generally, the GT2 tends to overestimate SC compared with Actigraph accelerometer measurements (MPE=−17.47%, mean difference [MD]=1318.82 steps). This overestimation is more pronounced in the MAAO group (MPE=−19.79%, MD=1516.62 steps) than in the YA group (MPE=−14.55%, MD=1068.63 steps). Furthermore, the error results (MPE=−17.47%, MAPE=25.77%) confirm the GT2’s validity for measuring SC in daily life. A independent *t* test comparing MAPE between the YA and MAAO groups indicates a significant difference (*t*=−3.45; *df*=571; *P*<.001), with the YA group showing a lower measurement error (MAPE=21.91%, SD=22.43%) than the MAAO group (MAPE=28.81%, SD=24.74). Finally, the Bland-Altman plot and Bull’s-eye diagrams (Figures1A  2) display minimal scatter bias, suggesting good overall consistency in GT2’s SC measurements. However, as the number of steps increases, the deviation becomes greater, implying a decrease in measurement consistency between the GT2 and the Actigraph (Figure 1A). Overall, the GT2 demonstrates good to excellent accuracy in SC measurement for both YA and MAAO groups in daily life settings.

### Total Daily Activity Energy Expenditure

First, Table 2 illustrates that TDAEE data measured by the GT2 and the accelerometer are correlated, evidenced by a PPMCC of 0.77 (*P*<.001; 95% CI 0.74-0.80) and an ICC of 0.75 (*P*<.001; 95% CI 0.67-0.80), indicating GT2’s moderate to good validity in measuring TDAEE. Second, Table 3 summarizes the agreements between the GT2 and Actigraph in terms of TDAEE during daily life in different groups. In daily settings, GT2 typically underestimates TDAEE (MPE=8.43%, MD=50.36 kcal) compared with the Actigraph accelerometer. The underestimation seems to be more frequent among MAAO adults (MPE=11.32%, MD=54.73 kcal) than among YAs (MPE=4.48%, MD=44.39 kcal). The error results (MPE=8.43%, MAPE=33.79%) suggest moderate validity of the GT2 for measuring TDAEE in daily life. A independent *t* test comparing MAPE between the YA and MAAO groups showed a significant difference (*t*=−6.70; *df*=512; *P*<.001), with the YA group exhibiting a lower measurement error (MAPE=30.32%, SD=21.48%) than the MAAO group (MAPE=36.29%, SD=22.68%). Finally, the Bland-Altman plot and Bull’s-eye diagrams (Figures1B  2) indicate moderate consistency in GT2’s TDAEE measurements, with a tendency to underestimate. In addition, the bias increases with increasing levels of TDAEE, suggesting that the error in GT2 measurements tends to amplify as activity energy expenditure increases (Figure 1B). Overall, the GT2 has moderate validity and may have limited measurement accuracy at high levels of activity energy expenditure.

### Total Sleep Time

First, Table 2 displays a significant correlation between the GT2 and the accelerometer, with a PPMCC of 0.47 (*P*<.001; 95% CI 0.40-0.54) and an ICC test score of 0.25 (*P*<.001; 95% CI −0.06 to 0.49). Second, we tested the difference between GT2 and Actigraph in terms of TST during daily life across different age groups, which is summarized in Table 3. The GT2 tends to underestimate TST (MPE=19.98%, MD=138.08 minutes) as compared with the Actigraph accelerometer measurements. The underestimation was evident between both YAs (MPE=22.13%, MD=156.33 minutes) and MAAO adults (MPE=18.35%, MD=123.15 minutes). The error results (MPE=19.98%, MAPE=23.29%) suggest moderate validity of the GT2 for measuring TST in daily life. A independent *t* test showed no significant difference in MAPE between the YA and MAAO groups (*t*=1.54; *df*=483; *P*=.12), indicating that the measurement error of the GT2 in the YA group (MAPE=24.39%, SD=13.84%) was comparable with that in the MAAO group (MAPE=22.45%, SD=13.58%). Finally, the Bland-Altman plots and Bull’s-eye diagrams (Figures1C  2) indicate that the GT2 tends to underestimate TST and that the underestimation increases as sleep time increases. Overall, when using the Actigraph GT3X+ as the criterion measure, the GT2 has poor to moderate validity in measuring TST among adults during daily life.

## Discussion

### Principal Findings

This study assessed the accuracy of the Huawei Watch GT2 in measuring SC, TDAEE, and TST under free-living conditions, while also exploring the validity differences between YAs and MAAO adults. The findings indicate that while the GT2 measures SC reasonably accurately, its accuracy in measuring TDAEE is lower, especially at higher levels of TDAEE. In addition, the accuracy of TST measurement appears to be lower when using Actigraph accelerometers as the reference criterion, highlighting the need for more valid standardized measures for further validation. Notably, the GT2 demonstrated higher accuracy in measuring SC and TDAEE in the YA group than in the MAAO group; however, the measurement errors for TST did not significantly differ between the 2 age groups.

For SC, the GT2 often reports higher counts than traditional accelerometers in daily scenarios (MD=1318 steps), yet it maintains a reasonable accuracy in SC, as evidenced by its high ICC value with accelerometer counts (ICC=0.88). This observation is in line with findings from prior research. For instance, Degroote et al [22] found that despite the Huawei watch’s tendency to overestimate SC (MD=1478 steps), its SC measurement accuracy remains robust, demonstrated by strong correlations with accelerometer data (ICC=0.88). The consistent overestimation observed across studies may be attributed to the differing placement of the devices [22,42]. Tudor-Locke et al [42] showed that wrist-attached devices tend to report higher SC than waist-attached devices in free-living conditions, with overestimations ranging from 2500 to 8700 steps per day. This phenomenon is particularly noticeable during sedentary and low-intensity intermittent activities, which are more prevalent than continuous walking in everyday life [43]. Consequently, while the GT2 accurately captures SC during regular walking (involving typical wrist and hip movements), it may also mistakenly count steps during wrist movements in nonwalking activities (eg, wrist oscillation without actual displacement), leading to higher counts than those recorded by waist-worn accelerometers. Further analysis through a independent *t* test on MAPE revealed that the GT2’s SC performance is superior in the YA group (MAPE=21.91%) compared with the MAAO group (MAPE=28.81%). This can be attributed to YAs’ more predictable movement patterns, which correspond more closely with the GT2’s step counting algorithm [43]. In addition, younger adults typically engage more in vigorous activities, making their movements more detectable than those of older adults, resulting in better accuracy for the GT2. This is in line with previous research indicating that consumer-grade activity trackers tend to be more accurate at moderate to vigorous walking speeds than at slower speeds [44]. Future studies may consider improving the calibration of SC algorithms in smartwatches to account for the variability in activity types and intensities, which may potentially enhance their accuracy across a wider range of movements.

To our knowledge, this is the first study to assess the GT2 smartwatch’s efficacy in measuring TDAEE in real-life settings. Consistent with findings from other smartwatch research on TDAEE [28,45-48], our results reveal that while the GT2 demonstrates moderate validity compared with the Actigraph, its accuracy in TDAEE measurements is still not ideal, as indicated by a high level of errors against the Actigraph (MAPE >30%). Previously, Le et al [27] analyzed the accuracy of GT2 compared with Cosmed K5 in measuring activity energy expenditure in outdoor scenarios and showed that the ICC was 0.76 and 0.68, and the MAPE was 9.9% and 11.9% in walking (6 km/hour, 2 km) and running (10 km/hour, 2 km) scenarios, respectively. These findings underscore the GT2’s moderate accuracy in activity energy expenditure measurement. Notably, our study extends the current findings by highlighting an increase in measurement error according to the rise in activity energy expenditure. Although the degree of error in our study appears higher than that reported in previous studies (eg, Le et al [27], Muggeridge et al [49], and Reddy et al [50]), we applied the device in a real-world setting over an extended period of 7 full days. It is important to consider that acceptable MAPE limits should be interpreted in the context of specific application scenarios and performance indicators. Previous research has shown that data collected in daily life are often less accurate than those generated under standardized, controlled, and relatively short-term conditions, such as in a laboratory setting [28,45,51]. This is because the accuracy of the device in real-world scenarios can be influenced by various factors, such as complex terrain and irregular activity patterns, which make it challenging to consistently identify such activities through acceleration counts [44,51]. Hence, scholars have clearly articulated the necessity of testing devices in free-living contexts, as they are designed to monitor daily living activities [52]. Again, this study emphasized that while the GT2 has good potentials for everyday health monitoring, users and researchers alike must remain cognizant of its limitations in accurately tracking TDAEE. The Huawei Watch GT2 may face challenges in accurately predicting energy expenditure in certain scenarios, especially when activity patterns are not very consistent. To address this, it is critical to further enhance sensor accuracy and better integrate multiple data sources, optimizing the algorithm’s ability to recognize and monitor activity patterns. The users should take the potential underestimation into consideration when interpreting the readings of their TDAEE. Such findings underscore the ongoing need for technological refinement and validation.

Likewise, the issue of the measurement error in TST by GT2 warrants further research attention. We identified an underestimation of TST (138.08 minutes) for the GT2, and a similar case is reported on Fitbit [53,54]. Compared with polysomnography, Meltzer et al [53] showed that the Fitbit Ultra (sensitive mode) underestimated TST by 105 minutes on average in youth, and Cook et al [54] also found that the Fitbit Flex (sensitive mode) underestimated TST (86.3 minutes) in individuals with unipolar major depressive disorder. The degree of TST underestimation of Fitbit seems smaller than what we found in GT2. However, the reference measure used in this study (the accelerometer) is different from that used in Fitbit validation studies (polysomnography). Polysomnography is the gold standard for TST measurement, whereas accelerometers often overestimate TST too. For instance, Zinkhan et al [55] found that Actigraph GT3X+ (hip) overestimated TST (81.1 minutes) as compared with polysomnography. Therefore, when hip-worn accelerometers are used as measurement criteria, the degree of underestimating TST by smartwatch is likely to be exaggerated [55,56]. It is worth noting that different wearing positions resulting in different displacement data may be one of the reasons why the accelerometer and GT2 data are not in agreement [42,55]. Specifically, during bed rest, wrist activity tends to be higher than hip activity, given that people may engage in activities involving use of wrists (eg, reading books or using a mobile phone); in fact, many participants orally reported habitual use of smartphones while lying in bed prior to falling asleep at night and getting up in the morning. In such cases, it is easy for smartwatch to capture wrist movements and accurately distinguish between awake and sleep states. However, hip-worn accelerometers are less able to monitor these activities and tend to recognize them as sleep time and therefore are more likely to overestimate sleep duration, especially in insomniacs [55]. As described in the reviews by Guillodo et al [57] and Evenson et al [45], the current evidence from reliability studies on sleep is still limited. Therefore, although results of this study suggest that the GT2 is likely to underestimate TST in reference to Actigraph, we should be cautious in drawing the conclusions. Future research is essential, and it should incorporate established standards such as polysomnography to validate the accuracy of smartwatch measurements for TST.

Smartwatches are becoming increasingly popular, but their use for monitoring and regulating health-related behaviors (eg, physical activity, sedentary behaviors, and sleep) is hindered by insufficient evidence of accuracy [23,28,29,31]. In particular, the accuracy of the Huawei Watch GT2, a highly popular wearable device, remains uncertain in terms of TDAEE and TST monitoring in daily life, and few studies have been conducted on large Asian populations [28,29,31]. Therefore, we focused on gathering empirical evidence on the GT2’s accuracy in the Chinese population, we believe such efforts are necessary and should be made on a regular basis. In this study, we found that the GT2 demonstrates good accuracy in measuring SC in adults during daily life but requires improvement in measuring TDAEE and TST. In addition, the accuracy of the GT2 in measuring SC and TDAEE may vary across different populations. Users should consider potential bias when evaluating activity goals based on GT2 measurements. This is particularly important to many users (especially those managing chronic diseases or aiming to improve physical health and well-being) who rely on the real-time feedback provided by smartwatches [58,59]. Future devices should better recognize users’ behavioral habits, so that the algorithms can be improved to better account for personal characteristics. Therefore, we call on mainstream smartwatch manufacturers to continuously improve their devices by introducing advanced techniques such as deep learning and sensor fusion to refine algorithms for more accurate health monitoring. Although it is challenging to establish a one-size-fits-all measurement standard due to individual and environmental variability, studies that consistently evaluate the validity of smartwatches can offer valuable feedback to improve algorithms, increase public recognition, and ultimately enhance their potential for health behavior management and promotion.

### Strengths and Limitations

This study has several strengths. First, the Huawei Watch is a widely used smartwatch with a high market share, especially in China (39%) [26], but only a few studies have provided empirical evidence of its accuracy [22,27]. The study examines the measurement accuracy of the GT2 in free-living settings. In particular, it is the first to examine the GT2’s accuracy in measuring TDAEE and TST in daily life, which are quite important parameters that have not received sufficient attention. Second, this study has a sample size that is relatively large among validation studies of smartwatches and covers a wide age distribution of participants (18-24 years in YAs; 55-91 years in MAAO adults), evidence of such studies is rarely reported before [30]. Collectively, the results supported the accuracy of this device in measuring SC and TDAEE in daily life. These findings may help users and researchers better determine how to use GT2 for practical and research purposes.

However, the results of this study should also be interpreted with consideration for several limitations. First, the proportion of female participants was higher in the MAAO group, and since we recruited a healthy population, the results may not be generalizable to populations with existing health conditions. Second, the reference measure of the study was research-grade accelerometers with known validity limitations, especially in terms of sleep measures. Future studies could use polysomnography and doubly labeled water methods to evaluate the accuracy of relevant parameters. Third, like other smartwatch validation studies, this study had access to only algorithmically processed data. In addition, as smartwatches are iteratively updated, the results may not generalize to newer models. Future studies should consider adopting more appropriate and feasible criterion measures based on the specific target behaviors to be assessed.

### Conclusions

In summary, the Huawei Watch GT2 serves as a valid measure of SC and shows moderate accuracy in measuring TDAEE. However, it should be used with caution for studies requiring high precision of TDAEE. Furthermore, the GT2 has been shown to underestimate TST measurements in reference to an accelerometer. Future studies should further validate its accuracy in monitoring TST using more valid methods, such as polysomnography. The GT2 demonstrated higher accuracy in measuring SC and TDAEE in the YA group compared with the MAAO group. However, the measurement errors for TST did not differ significantly between the 2 age groups. As smartwatches are constantly updated, their accuracy should be regularly evaluated in various settings and populations to ensure proper application in health-related behavior monitoring and promotion.

## Abbreviations

ICC: intraclass correlation coefficient
MAAO: middle-aged and older
MAPE: mean absolute percentage error
MD: mean difference
MPE: mean percentage error
PPMCC: Pearson product-moment correlation coefficient
SC: step count
TDAEE: total daily activity energy expenditure
TST: total sleep time
YA: young adult
